# Long-Term Outcomes of Aortic Stenosis Patients with Different Flow/Gradient Patterns Undergoing Transcatheter Aortic Valve Implantation

**DOI:** 10.3390/jcm13051200

**Published:** 2024-02-20

**Authors:** George Oikonomou, Anastasios Apostolos, Maria Drakopoulou, Chryssavgi Simopoulou, Maria Karmpalioti, Pantelis Toskas, Konstantinos Stathogiannis, Maria Xanthopoulou, Nikolaos Ktenopoulos, George Latsios, Andreas Synetos, Constantinos Tsioufis, Konstantinos Toutouzas

**Affiliations:** First Department of Cardiology, National and Kapodistrian University, “Hippokration” General Hospital of Athens, 11527 Athens, Greece; geooik88@gmail.com (G.O.); anastasisapostolos@gmail.com (A.A.); mdrakopoulou@hotmail.com (M.D.); chryssa_1991@hotmail.com (C.S.); karmpaliotimaria@gmail.com (M.K.); toskas.pad@gmail.com (P.T.); kstathog@hotmail.com (K.S.); xanthopoulou11maria@gmail.com (M.X.); nikosktenop@gmail.com (N.K.); glatsios@gmail.com (G.L.); synetos@yahoo.com (A.S.); ktsioufis@gmail.com (C.T.)

**Keywords:** flow, gradient, aortic stenosis, transcatheter aortic valve implantation, mortality, long term, left ventricular ejection fraction

## Abstract

**Background:** Few data exist on the comparative long-term outcomes of severe aortic stenosis (AS) patients with different flow-gradient patterns undergoing transcatheter aortic valve implantation (TAVI). This study sought to evaluate the impact of the pre-TAVI flow-gradient pattern on long-term clinical outcomes after TAVI and assess changes in the left ventricular ejection fraction (LVEF) of different subtypes of AS patients following TAVI. **Methods:** Consecutive patients with severe AS undergoing TAVI in our institution were screened and prospectively enrolled. Patients were divided into four subgroups according to pre-TAVI flow/gradient pattern: (i) low flow—low gradient (LF-LG): stroke volume indexed (SVi) ≤ 35 mL/m^2^ and mean gradient (MG) < 40 mmHg); (ii) normal flow—low gradient (NF-LG): SVi > 35 mL/m^2^ and MG < 40 mmHg; (iii) low flow—high gradient (LF-HG): Svi 35 mL/m^2^ and MG ≥ 40 mmHg and (iv) normal flow—high gradient (NF-HG): SVi > 35 mL/m^2^ and MG ≥ 40 mmHg. Transthoracic echocardiography was repeated at 1-year follow-up. Clinical follow-up was obtained at 12 months, and yearly thereafter until 5-year follow-up was complete for all patients. **Results:** A total of 272 patients with complete echocardiographic and clinical follow-up were included in our analysis. Their mean age was 80 ± 7 years and the majority of patients (N = 138, 50.8%) were women. 62 patients (22.8% of the study population) were distributed in the LF-LG group, 98 patients (36%) were LF-HG patients, 95 patients (34.9%) were NF-HG, and 17 patients (6.3%) were NF-LG. There was a greater prevalence of comorbidities among LF-LG AS patients. One-year all-cause mortality differed significantly between the four subgroups of AS patients (log-rank p: 0.022) and was more prevalent among LF-LG patients (25.8%) compared to LF-HG (11.3%), NF-HG (6.3%) and NF-LG patients (18.8%). At 5-year follow-up, global mortality remained persistently higher among LF-LG patients (64.5%) compared to LF-HG (47.9%), NF-HG (42.9%), and NF-LG patients (58.8%) (log-rank p: 0.029). At multivariable Cox hazard regression analysis, baseline SVi (HR: 0.951, 95% C.I.; 0.918–0.984), the presence of at least moderate tricuspid regurgitation at baseline (HR: 3.091, 95% C.I: 1.645–5.809) and at least moderate paravalvular leak (PVL) post-TAVI (HR: 1.456, 95% C.I.: 1.106–1.792) were significant independent predictors of late global mortality. LF-LG patients and LF-HG patients exhibited a significant increase in LVEF at 1-year follow-up. A lower LVEF (*p* < 0.001) and a lower Svi (*p* < 0.001) at baseline were associated with LVEF improvement at 1-year. **Conclusions:** Patients with LF-LG AS have acceptable 1-year outcomes with significant improvement in LVEF at 1-year follow-up, but exhibit exceedingly high 5-year mortality following TAVI. The presence of low transvalvular flow and at least moderate tricuspid regurgitation at baseline and significant paravalvular leak post-TAVI were associated with poorer long-term outcomes in the entire cohort of AS patients. The presence of a low LVEF or a low SVi predicts LVEF improvement at 1-year.

## 1. Introduction

Based on the current European and American guidelines, severe aortic stenosis (AS) is defined as an effective aortic valve area (AVA) of <1 cm^2^ (or indexed for body surface area [BSA], AVA/BSA < 0.6 cm^2^/m^2^) and a mean pressure gradient (MPG) and peak velocity (Vmax) of ≥40 mmHg and ≥4.0 m/s, respectively [1,2]. However, in clinical practice, there is frequently an inconsistency in diagnostic criteria in patients in whom AS appears to be severe based on AVA but moderate or even mild based on transvalvular gradients [3,4,5,6,7]. This inconsistency is caused by a reduced left ventricular (LV) stroke volume, which leads to a reduction of transaortic flow and gradient. A low-flow, low-gradient (LF-LG) severe AS in relation to a decrease in left ventricular ejection fraction (LVEF) (i.e., LVEF < 50%) (low EF, LF-LG AS) may be observed in approximately 5% to 10% of patients with severe AS [8,9]. Conservative management of these patients has been associated with a dismal prognosis, with survival rates < 70% and <50% at 1- and 3-year follow-up, respectively [10]. In the last years, a second type of LF-LG AS patients has been recognised with low-flow conditions caused by a decreased stroke volume due to a small LV cavity size and restrictive physiology [5]. This phenomenon has been described as “paradoxical” low-flow, low-gradient AS (PLF-LGAS), as it might be observed despite a preserved EF (LVEF ≥ 50%) in approximately 10–25% of patients with severe AS [7,11,12,13,14]. In patients with LFLG-AS, surgical aortic valve replacement (SAVR) has been associated with a significant improvement in mid- to long-term survival, but operative mortality remains high (6% to 30%) [9,15,16,17].

Transcatheter aortic valve implantation (TAVI) has revolutionized the treatment of severe AS, since its introduction into clinical practice as an alternative option to surgical aortic valve replacement (SAVR) in intermediate and high to prohibitive-risk patients [18,19,20,21,22]. In the last years, numerous technological refinements and the growing experience have expanded the indications for TAVI towards the treatment of younger, lower-risk patients [23,24]. The increased number of AS patients, who are appropriate candidates for TAVI has created a wide spectrum of AS patients with different phenotypes, in which TAVI’s long-term safety and efficacy have not yet been well investigated. Recent observational studies have suggested that TAVI represents an alternative treatment strategy to SAVR for treating patients with LFLG-AS [25,26,27,28,29,30,31,32,33,34]. Yet, these data are limited by their retrospective nature and relatively short duration of follow-up. Hence, an ongoing debate exists concerning the therapeutic advantages of TAVI across the various AS phenotypes based on flow/gradient patterns. The aim of our study was to assess late clinical outcomes and their contributing factors as well as LVEF changes over time following TAVI in the entire spectrum of AS patients based on the baseline flow/gradient pattern.

## 2. Methods

### 2.1. Study Population

All patients with severe symptomatic AS referred for TAVI in our institution between 1 January 2015 and 31 December 2018 were screened for inclusion in our study. Patient enrollment and data collection started in January 2015 and 302 patients were prospectively enrolled until December 2018. Echocardiograms at baseline and one year after TAVI were acquired for longitudinal data analysis. Patients were followed up by clinical visits or phone contact at 12 months after TAVI and yearly thereafter until all patients completed the 5-year follow-up. Only patients with available clinical and echocardiographic data were included in the study. Indication for TAVI was based on the assessment of the Heart Team of our institution, taking into account clinical, anatomical, and echocardiographic characteristics according to the guidelines. All participants underwent comprehensive physical examination, coronary angiography, and multi-slice computed tomography (MSCT) before TAVI. Informed consent was obtained from all subjects involved in the study. The study was conducted according to the guidelines of the Declaration of Helsinki, and approved by the Institutional Review Board of our institution (Hippokration General Hospital of Athens). A total of 30 patients were excluded due to missing clinical follow-up data or unavailable echocardiograms. Self-expandable transcatheter heart valves were implanted in all patients. Transfemoral access was the preferred access, whereas subclavian access was used only in patients with inappropriate iliofemoral anatomy.

### 2.2. Clinical Data

Baseline demographic characteristics (sex, gender, age, and body mass index), comorbidities (diabetes mellitus, hypertension, coronary artery disease (CAD), chronic obstructive pulmonary disease (COPD), peripheral arterial disease (PAD), chronic kidney disease (CKD), history of myocardial infarction (MI), previous cardiac interventions; percutaneous coronary intervention (PCI) or coronary artery bypass graft surgery (CABG), Euroscore II (European system for cardiac operative risk evaluation) and NYHA (New York Heart Association) functional class were recorded. The presence of CAD was defined as a history of percutaneous coronary intervention, surgical treatment, or the presence of angiographically significant stenosis (>50%) in at least one epicardial coronary artery. CKD was defined according to Kidney Disease Improving Global Outcomes (KDIGO) criteria.

### 2.3. Doppler Echocardiography

All patients underwent comprehensive echocardiographic examination pre-TAVI and at 1-year post-TAVI. Echocardiographic parameters included the following variables: mean transvalvular gradient, peak aortic jet velocity (Vmax), aortic jet velocity time integral (VTI), LV outflow tract (LVOT) diameter, LVOT VTI, AVA and stroke volume (SV). AVA was estimated using the continuity equation. SV was calculated using the cross-sectional area of the LVOT and VTI of the LVOT flow as follows: SVLVOT = (cross-sectional area LVOT) × VTILVOT. VTILVOT was acquired by the pulsed wave Doppler technique. Stroke volume index (SVi) was measured using the following equation: SV/BSA (Body Surface Area). LVEF was measured by the biplane Simpson method.

Severe AS is defined as an effective aortic valve area (AVA) of <1 cm^2^ (or indexed for [BSA], AVA/BSA < 0.6 cm^2^/m^2^) a mean pressure gradient (MPG) of ≥40 mmHg and peak aortic velocity (Vmax) of >4.0 m/s. Low-flow (LF) was defined as an LV stroke volume index (SVi) ≤ 35 mL/m^2^, normal flow (NF) as SVi > 35 mL/m^2^, low-gradient (LG) as a mean transvalvular gradient < 40 mm Hg, and high gradient (HG) as a mean gradient ≥ 40 mmηg^2^. Accordingly, based on transvalvular flow (F) and gradient (G) we have divided our patients into four distinct subgroups; low-flow, high-gradient (LF-HG), low-flow, low-gradient (LF-LG), normal-flow, high-gradient (NF-HG) and normal-flow, low-gradient (NF-LG) (Table 1).

### 2.4. Study Endpoints

The primary endpoints were: (1) early (1-year) and late (5-year) all-cause mortality and changes in LVEF from baseline to 1-year follow-up. Secondary endpoints were 1-year and 5-year cardiovascular mortality, 1-year MACCE (Major Adverse Cardiovascular and Cerebrovascular Events) rates, periprocedural events (permanent pacemaker implantation, major vascular complications, major bleeding complications at 30 days following TAVI), and factors associated with increased late global mortality and LVEF improvement at 1-year. MACCE is a composite outcome, which includes all-cause mortality, non-fatal MI, and non-fatal stroke. The definition and the evaluation of clinical events were performed according to VARC-2 (Valve Academic Research Consortium—(2)) criteria. Bleeding events were considered major, when they were categorized as grade 3–5, according to Bleeding Academy Research Consortium (BARC).

### 2.5. Statistical Analysis

Continuous data were expressed as mean ± SD or median (interquartile range) and were tested for the normality of distribution with the Shapiro-Wilk test. Categorical data were expressed as numbers and percentages. Patients were compared with the Student’s *t*-test or the Wilcoxon rank sum test for continuous variables and with the chi-square test or Fisher exact test for categorical variables, as appropriate. Kaplan-Meier curves and log-rank test of the time-to-event data were used to evaluate global and cardiovascular mortality. The association between baseline clinical, echocardiographic variables, and periprocedural variables with global mortality was assessed with the use of Cox proportional hazard analyses. Variables with a *p*-value < 0.10 in univariable analysis were entered into the multivariable model. A paired samples *t*-test was used to analyze the changes in LVEF over time. The predictors of improvement in LVEF at 1-year follow-up were determined using a linear regression analysis. All *p*-values < 0.05 were considered statistically significant. Statistical analysis was performed using the statistical package SPSS (version 26, SPSS Inc., Chicago, IL, USA).

## 3. Results

Between 1 January 2015 and 31 December 2018, a total of 302 patients undergoing TAVI in our center were screened and considered eligible for inclusion in the study. Completed echocardiographic and clinical follow-up, up to five years was available in 272 patients, who were included in the analysis, accounting for approximately 10% loss to follow-up. Baseline demographic and clinical characteristics are presented comprehensively in Table 2. The mean age was 80 ± 7 years and 50.8% were women. The median Euroscore II was 5.14 {interquartile range (IQR): 4.22–6.48}. LF-LG AS was present in 62 patients, while 95 patients presented with NF-HG AS, 98 patients had LF-HG AS and 17 patients presented with NF-LG AS. LF-LG AS patients had a higher prevalence of previous myocardial infarction (MI) (*p* = 0.005) and showed a trend towards a greater prevalence of comorbidities such as peripheral artery disease and at least moderate mitral regurgitation. This was reflected by the significantly higher Euroscore II in the LF-LG group compared with the other groups of patients (*p* = 0.004 for between-group difference). The history of CABG was more common among LG compared to HG patients.

Echocardiographic and MSCT-derived variables were compared between the different groups of AS patients. Baseline LVEF, mean gradient, AVA, and SVi were significantly lower among LF-LG patients (all *p* < 0.001). There was no significant difference in the pre-TAVI pulmonary artery systolic pressure between groups. The rate of severe aortic valve (AV) calcification assessed semi-quantitatively by MSCT was also comparable between groups (Table 3).

### 3.1. One-Year and Late Clinical Outcomes

The one-year clinical outcomes are shown in Table 4. One-year all-cause mortality was significantly higher in LF-LG patients compared to NF-HG patients (25.8% vs. 6.3%, respectively, *p* = 0.011) but not compared to NF-LG (25.8% vs. 18.8%, respectively, *p* = 0.729) or LF-HG patients (25.8% vs. 11.5%**,** respectively, *p* = 0.084) (Table 4). The median follow-up duration was 59 months (IQR: 31 to 81 months) and the late cumulative clinical outcomes are shown in Table 4. A total of 137 patients died at five-year follow-up, leading to a global cumulative mortality of 50.36%. Death was from cardiovascular causes in 75 patients (27.57%). Five-year all-cause mortality was significantly higher in LF-LG patients compared to NF-HG patients (64.5% vs. 42.5%, respectively, *p* = 0.038) but not compared to NF-LG patients (64.5% vs. 58.8%, respectively, *p* = 1.00) and LF-HG patients (64.5% vs. 47.9%, respectively, *p* = 0.152). This was confirmed by the multivariable Cox proportional hazard regression analysis, where the presence of LF-LG AS was independently associated with increased late all-cause mortality (HR: 1.757, *p* = 0.018) with NF-HG AS being the reference standard.

**Figure 1:**
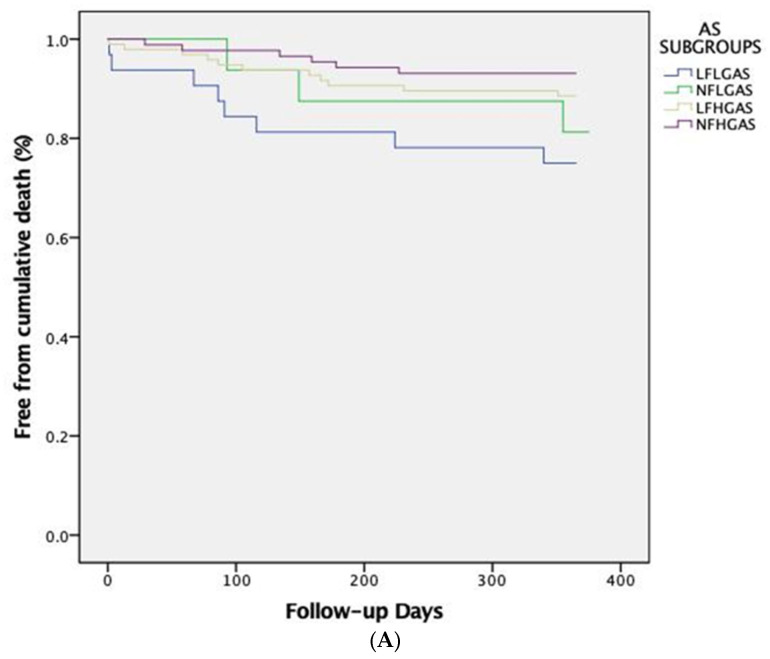

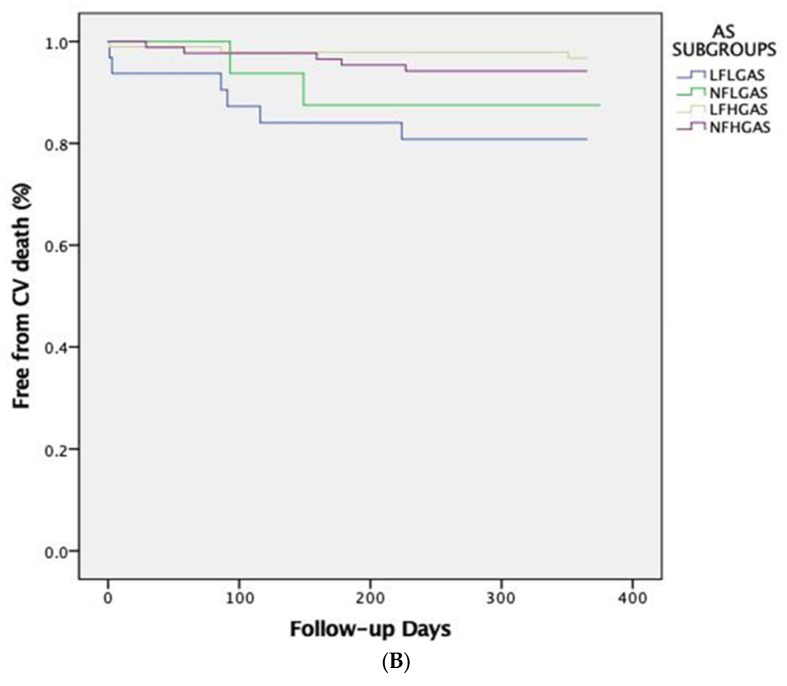
Kaplan-Meier survival curves at 1-year follow-up showing (**A**) global and (**B**) cardiovascular mortality across different AS subgroups based on pre-TAVI flow/gradient status. AS; Aortic Stenosis, LFLG; Low Flow-Low Gradient, NFLG; Normal Flow-Low Gradient, LFHG; Low Flow-High Gradient, NFHG; Normal Flow-High Gradient, CV; Cardiovascular.

**Figure 2:**
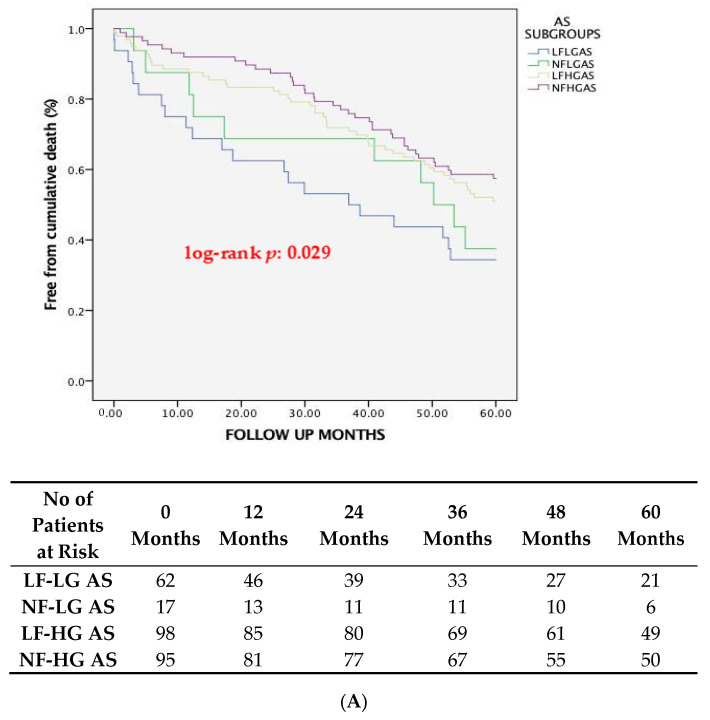

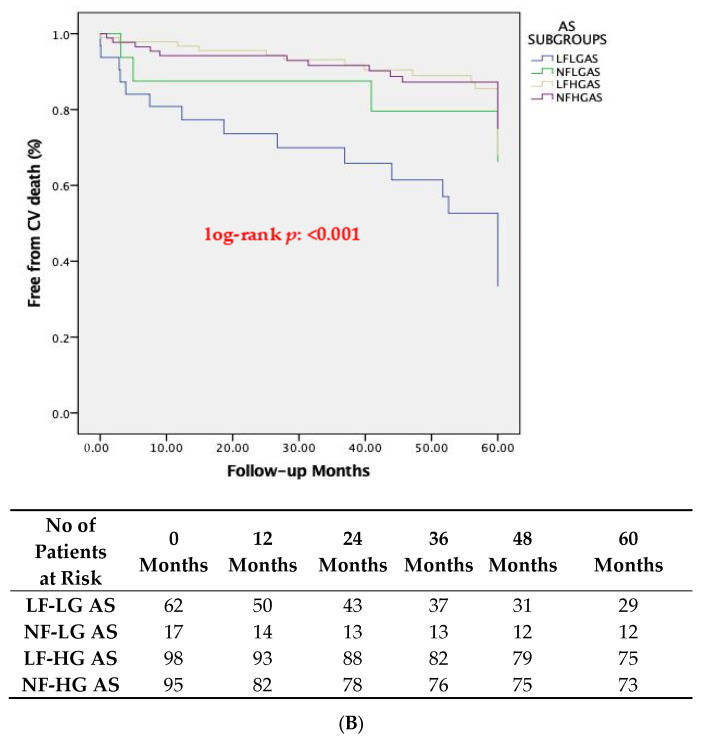
Kaplan-Meier survival curves at 5-year follow-up showing (**A**) global and (**B**) cardiovascular mortality across different AS subgroups based on pre-TAVI flow/gradient status. AS, Aortic Stenosis; LF-LG, Low Flow-Low Gradient; NF-LG, Normal Flow-Low Gradient; LF-HG, Low Flow-High Gradient; NF-HG, Normal Flow-High Gradient; CV, Cardiovascular.

One-year cardiovascular mortality differed significantly between the four subgroups of AS patients (*p* = 0.018), which was driven primarily by the significantly higher cardiovascular mortality of LF-LG compared to LF-HG patients (19.3% vs. 3.1%, respectively, *p* = 0.008). At 5-year follow-up, cardiovascular mortality was significantly higher in LF-LG patients (53.2%), compared to LF-HG (21.4%, *p* = 0.001) and NF-HG patients (17.9%, *p* = 0.001) but not compared to NF-LG patients (23.5%, *p* = 0.075). The Kaplan-Meier curves for global and cardiovascular mortality at 1-year and 5-year follow-ups are shown in Figure 1A,B and Figure 2A,B, respectively.

Incidence of one-year MACCE (a composite endpoint of death, non-fatal MI, non-fatal stroke) was significantly higher in patients with LF-LG pattern, compared to NF-HG patients (25.8% vs. 8.4%, respectively, *p* = 0.029), but was comparable to NF-LG and LF-HG patients (*p* = 1.00 and *p* = 0.092, respectively). No statistically significant differences between groups were observed regarding the rates of periprocedural events (permanent pacemaker implantation, major vascular complications, and life-threatening/major bleeding complications at 30 days following TAVI) (Table 5).

### 3.2. Predictors of Late Global Mortality

The factors associated with long-term all-cause mortality in univariate analysis were included in the multivariate model (Table 6). In a multivariable Cox proportional hazards model, the independent predictors of late global mortality after TAVI in the entire cohort were transvalvular flow and the presence of at least moderate tricuspid regurgitation at the baseline echocardiographic evaluation (HR: 3.091, 95% CI: 1.645–5.809, *p* < 0.001) as well as the presence of moderate or greater post-TAVI paravalvular leak (PVL) (HR: 1.456, 95% CI: 1.106–1.792, *p* = 0.042).

### 3.3. Impact of Pre-Tavi Flow on Late Outcomes

**Figure 3:**
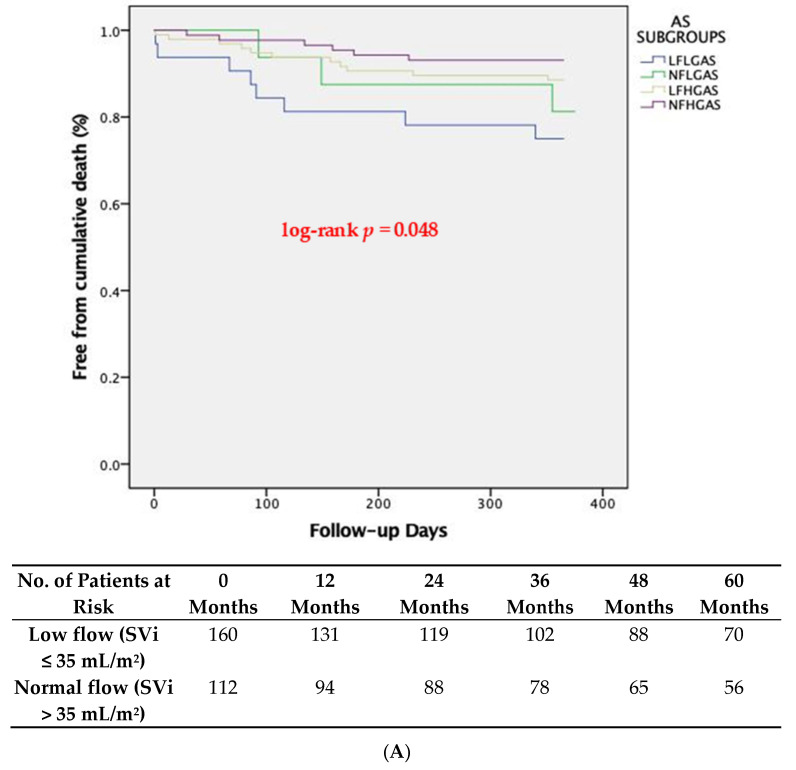

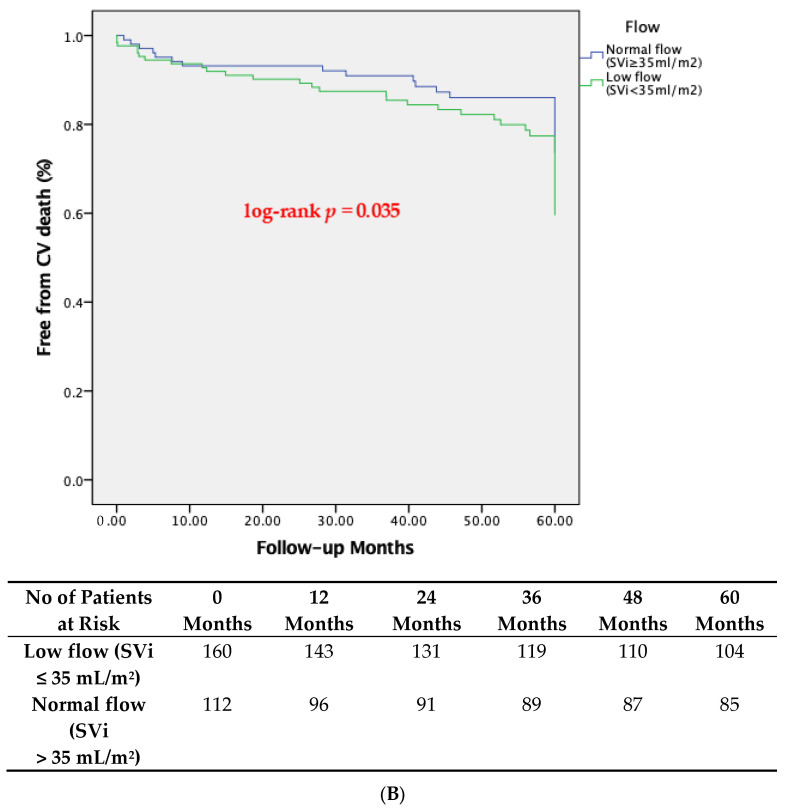
Kaplan-Meier survival curves at 5-year follow-up showing (**A**) global and (**B**) cardiovascular mortality stratified based on pre-TAVI flow status. CV; Cardiovascular.

Patients who exhibited low flow pre-procedurally by means of the Doppler-based estimation of SVi had increased 5-year all-cause and cardiovascular mortality compared to patients with normal flow, which is depicted in the respective Kaplan-Meier survival curves (Figure 3A,B, respectively). This was confirmed in the multivariable Cox proportional hazard regression analysis, where SVi emerged as a significant independent predictor of late global mortality after TAVI (HR:0.951 per unit increase of SVi, *p* = 0.004) (Table 6).

### 3.4. Changes in LVEF over Time

**Figure 4:**
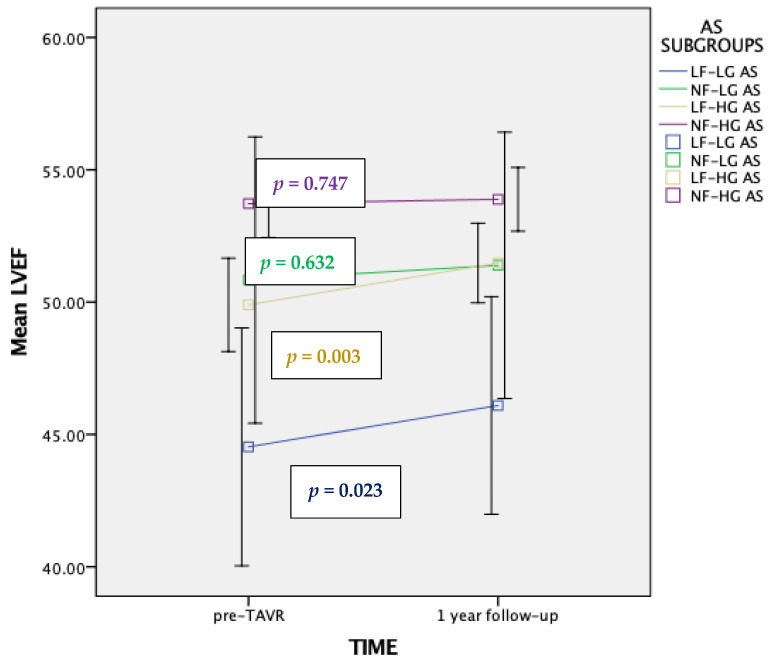
Changes in LVEF over time across different AS subgroups according to pre-TAVI flow/gradient status. AS; Aortic Stenosis, LVEF; Left Ventricular Ejection Fraction, LF-LG; Low Flow-Low Gradient, NF-LG; Normal Flow-Low Gradient, LF-HG; Low Flow-High Gradient, NF-HG; Normal Flow-High Gradient.

A total of 282 patients had an echocardiographic examination at 1-year follow-up (93.3% of the initially enrolled population) and all of them had a baseline echocardiogram for comparison. Among the 272 patients included in the final analysis, 70 patients (25.7%) exhibited some degree of improvement in LVEF at follow-up (mean increase of 3.23%; 95% C.I.: 1.67% to 4.83%, *p* < 0.001). Patients in the LF-LG and LF-HG subgroups had a statistically significant increase in LVEF over time (*p* = 0.023 and *p* = 0.003, respectively), while in NF-LG and NF-HG patients no impact was observed on their LVEF 1-year after TAVI (Figure 4).

In multivariable linear regression analysis, the factors independently associated with LVEF improvement at 1-year follow-up was the presence of a lower LVEF at baseline (OR: 0.906, 95% CI: 0.873–0.940, *p* < 0.001, per unit increase of baseline LVEF) and the presence of a lower SVi at baseline (OR:0.898, 95% CI: 0.864–0.931, *p* < 0.001, per unit increase of SVi) (Table 7).

## 4. Discussion

The evolving clinical indications for TAVI have been recently illustrated by the results of several randomized trials, collectively showing TAVI to represent a valid alternative to SAVR for patients with intermediate to high operative risk [35]. Recent observational studies dedicated to LF-LG AS patients have outlined favorable early outcomes following TAVI [36] with lower 30-day mortality rates compared to those reported in prior TAVI studies evaluating LF-LG AS patients [31,37] as well as SAVR studies in LF-LG AS, despite including younger and lower risk patients [9,10,16,17,37,38,39,40,41,42]. However, despite the good early results post-TAVI in LF-LG AS patients and the constant optimization of the final hemodynamic result achieved with the latest THVs (transcatheter heart valves), the mid-term all-cause mortality remains high with approximately one-third of LF-LG patients dying after a median follow-up of 2-years [43]. Our study wished to expand on that knowledge and investigate whether this rise in all-cause mortality in LF-LG AS patients persists up to 5-years of follow-up and holds statistical significance over the other subgroups of AS patients.

Our study confirms the relatively high one-year mortality among LF-LG AS patients, albeit slightly lower than that reported in previous TAVI studies on LF-LG AS patients, as approximately one-fourth of LF-LG patients had died at 1-year following TAVI. Namely, Lauten et al. found that patients with low flow and impaired LV function had a significantly higher mortality within 1-year following TAVI compared to HG AS patients (32.3% vs. 19.8%, *p* = 0.001), while patients with low flow and preserved LV function had comparable 1-year mortality rates to HG AS patients (*p* = 0.192) [33]. In addition, Baron et al. found similarly high mortality rates among patients with low aortic valve gradients (AVG) and LV dysfunction during the first year following TAVI [37]. In our study, the excess in global mortality among LF-LG patients can be seen throughout the follow-up period up to 5-years [33,37]. In particular, LF-LG patients exhibited greater 5-year all-cause as well as cardiovascular mortality rates compared to NF-HG AS patients, while there was a trend towards greater long-term all-cause mortality compared to LF-HG and NF-LG patients.

The main factors associated with poorer long-term outcomes after TAVI were the presence of a low transvalvular flow and at least moderate tricuspid regurgitation at the baseline echocardiographic evaluation and the presence of at least moderate paravalvular leak following TAVI. Of note, our study adds to the existing published reports in several ways. First of all, in line with the staging classification of the extent of cardiac damage associated with AS proposed by Genereux et al., our findings add to the prognostic value of tricuspid regurgitation, the latter being a marker of advanced cardiac damage in AS patients [44]. Of note, we have shown that tricuspid regurgitation is a powerful predictor of long-term all-cause mortality after TAVI beyond 1-year. Second, our finding that LV dysfunction was not independently associated with long-term mortality after adjusting for flow status and other factors is in line with the results in the Baron et al. [38] study and emphasizes the benefits of TAVI, even in patients with severe LV dysfunction. Our finding that low transvalvular flow, but not reduced LVEF was associated with increased long-term all-cause mortality could be explained by the intrinsic myocyte dysfunction related to a low-flow status. Previous studies have demonstrated that patients with low-flow, low-gradient AS have evidence of myocardial fibrosis [45], a finding that has been linked to abnormal LV remodeling and reduced compliance and filling of the LV [14]. In fact, in some studies, low SVi has been shown to be a more powerful independent predictor of post-TAVI mortality than either LVEF or AVG [31]. From a practical perspective, our findings suggest that the presence of low flow and/or significant TR may identify a cohort of AS patients, who derive less long-term benefit from TAVI and are in need of close surveillance following TAVI. Nevertheless, it is important to recognize that neither low-flow status nor TR regurgitation identifies a group of patients with sufficiently poor outcomes to preclude consideration for TAVI, in the absence of other indicators of poor prognosis, but should rather be considered as markers of extensive cardiac damage, necessitating a closer follow-up, appropriate management of comorbidities and implementation of guideline-directed optimal medical therapy following TAVI. Finally, our study confirms the incremental prognostic value of PVL even at 5-years following TAVI, highlighting the need to achieve good sealing with the transcatheter heart valve, especially in patients with an already impaired LV with poor reserve. However, if significant PVL cannot be avoided, these patients should be closely monitored and treated promptly with percutaneous PVL closure, since it has been shown to lead to sustained improvements in clinical outcomes [46].

Some studies suggested that TAVI may be associated with enhanced and more rapid recovery of LV function compared with SAVR especially among patients with depressed systolic function, and this could be of major importance in patients with LFLG-AS [32,34] Indeed, our study approximately 50% of LF-LG patients improved their LVEF at 1-year follow-up and there was a greater improvement in LVEF at 1-year among LF-LG and LF-HG patients compared to NF-LG and NF-HG patients. In fact, LVEF recovery at 1-year follow-up was predicted by a lower baseline LVEF and stroke volume index, which could probably leave more space for improvement over time.

## 5. Limitations

Our study has several limitations that should be addressed. Firstly, this was a single-center study. Whereas most patients were included in this study prospectively, data were collected retrospectively in about one-half of the patients. The study had no onsite monitoring or event adjudication committee. This was partially compensated by remote electronic data monitoring to actively search and correct missing and/or inconsistent information, including a thorough evaluation of the causes of mortality. In addition, no centralized echocardiography core lab analysis was performed on the echocardiographic data. We acknowledge that the number of primary endpoint events was small to allow for the generalization of our findings. Finally, we could not systematically collect and provide DSE (dobutamine stress echocardiography) data on LF-LG patients with low LVEF and assess its prognostic relevance.

## 6. Conclusions

Patients with LF-LG AS have acceptable 1-year outcomes with significant improvement in LVEF at 1-year follow-up but exhibit exceedingly high 5-year mortality following TAVI. The presence of low transvalvular flow and at least moderate tricuspid regurgitation at baseline and significant paravalvular leak post-TAVI were associated with poorer long-term outcomes in the entire cohort of AS patients. The presence of a low LVEF or a low SVi predicts LVEF improvement at 1-year.

## Figures and Tables

**Table 1:** Subgroup definitions.

Groups	Mean Transvalvular Gradient	Stroke Volume Index
Low-flow, high-gradient (LF-HG)	≥40 mm Hg	≤35 mL/m^2^
Low-flow, low-gradient (LF-LG)	<40 mm Hg	≤35 mL/m^2^
Normal-flow, high-gradient (NF-HG)	≥40 mm Hg	>35 mL/m^2^
Normal-flow, low-gradient (NF-LG)	<40 mm Hg	>35 mL/m^2^

**Table 2:** Baseline demographic and clinical characteristics of the study population.

	All Patients (N = 272)	NF-HG AS 95 (34.9%)	LF-HG AS 98 (36%)	NF-LG AS 17 (6.3%)	LF-LG AS 62 (22.8%)	*p* Value
**Age, yrs**	80 ± 7	80 ± 7	81 ± 7	77 ± 8	78 ± 9	0.124
**BMI, kg/m^2^**	26.06 ± 3.17	25.32 ± 2.75	26.83 ± 3.48	26.54 ± 3.64	25.67 ± 2.56	0.008
**Male, (%)**	134 (49.2%)	34 (35.8%)	50 (51%)	9 (52.9%)	41(65.6%)	0.018
**Diabetes, (%)**	87 (32%)	32 (33.7%)	29 (29.6%)	7 (41.2%)	19 (31.3%)	0.623
**Hypertension, (%)**	206 (75.7%)	67 (70.5%)	76 (77.6%)	11 (64.7%)	52 (84.4%)	0.475
**CAD, (%)**	129 (47.4%)	41 (43.2%)	44 (44.9%)	9 (52.9%)	35 (56.3%)	0.268
**Previous MI, (%)**	55 (20.2%)	18 (18.9%)	15 (15.3%)	3 (17.6%)	19 (31.3%)	0.005
**Previous PCI, (%)**	36 (13.2%)	11 (11.2%)	11 (10.8%)	2 (14.3%)	12 (19.2%)	0.689
**Previous CABG, (%)**	63 (23.2%)	12 (12.4%)	24 (25.3%)	6 (35.7%)	21(34.6%)	0.025
**CKD, (%)**	114 (41.9%)	36 (37.9%)	42 (42.9%)	5 (29.4%)	31 (50%)	0.469
**PAD, (%)**	100 (36.7%)	29 (30.5%)	30 (30.6%)	4 (23.5%)	37 (59.4%)	0.063
**COPD, (%)**	71 (26.1%)	22 (23.2%)	25 (25.5%)	7 (41.2%)	17 (28.1%)	0.694
**EUROSCOREII**	5.14 (4.22–6.48)	4.50 (3.42–6.0)	5.15 (4.60–6.48)	5.62 (4.95–6.88)	8.34 (4.84–13.58)	0.004
**NYHA III/IV, (%)**	265 (97.4%)	92 (97.9%)	96 (98%)	17 (100)%	60 (96.9%)	0.850

BMI; Body Mass Index, CABG; Coronary Artery Bypass Graft surgery, CAD; Coronary Artery Disease, CKD; Chronic Kidney Disease COPD; Chronic Obstructive Pulmonary Disease, HG; High-Gradient, LF; Low-Flow, LG; Low-Gradient, MI; Myocardial Infarction; NF; Normal Flow, NYHA; New York Heart Association, PAD; Peripheral Arterial Disease, PCI; Percutaneous Coronary Interventions. Euroscore II is presented as median value {interquartile range}. Categorical variables are expressed as absolute numbers and percentages N (%), and continuous variables as mean ± SD.

**Table 3:** Baseline echocardiographic and MSCT variables of the study population.

	All Patients (N = 272)	NFHGAS (N = 95)	LFHGAS (N = 98)	NFLGAS (N = 17)	LFLGAS (N = 62)	*p* Value
**LVEF, %**	51 ± 9	54 ± 6	50 ± 9	50 ± 11	44 ± 12	<0.001
**Mean gradient, mmHg**	50 ± 15	57 ± 14	53 ± 11	34 ± 6	32 ± 9	<0.001
**AVA, cm^2^**	0.61 ± 0.15	0.68 ± 0.14	0.52 ± 0.11	0.77 ± 0.11	0.59 ± 0.16	<0.001
**Moderate-severe MR pre-TAVI**	86 (31.6%)	19 (20.3%)	34 (35%)	7 (38.5%)	26 (42.9%)	0.069
**Moderate-severe TR pre-TAVI**	64 (23.5%)	19 (20%)	28 (28.6%)	5 (29.4%)	12 (19.3%)	0.438
**Stroke volume indexed, mL/m^2^**	35 ± 11	45 ± 9	29 ± 6	40 ± 4	26 ± 5	<0.001
**Pulmonary artery systolic pressure (PASP) (mmHg)**	44.43 ± 12.50	44.95 ± 12.52	44.98 ± 13.46	41.76 ± 8.46	42.61 ± 11.22	0.620
**Severe AV calcification based on MSCT**	172 (63.2%)	58 (61.4%)	62 (63.2%)	14 (80%)	38 (61.1%)	0.717

LVEF; Left Ventricular Ejection Fraction, AVA; Aortic Valve Area, MR; Mitral Regurgitation, TAVI; Transcatheter Aortic Valve Implantation, TR; Tricuspid Regurgitation, AV; Aortic Valve, MSCT; Multi-Slice Computed Tomography. Categorical variables are expressed as absolute numbers and percentages, continuous as Mean ± SD.

**Table 4:** Primary endpoints: 1-year and 5-year all-cause mortality rates. Secondary endpoints: 1-year and 5-year CV mortality rates, 1-year MACCE rates. Values are N (%).

	LF-LG AS (N = 62)	NF-LG AS (N = 17)	LF-HG AS (N = 98)	NF-HG AS (N = 95)	p LF-LG AS vs. NF-LG AS	p LF-LG AS vs. LF-HG AS	p LF-LG AS vs. NF-HG AS	*p* *
1-year all-cause mortality, N (%)	16 (25.8)	3 (18.8)	11 (11.5)	6 (6.3)	0.729	0.084	0.011	0.048
1-year CV mortality, N (%)	12 (19.3)	2 (12.5)	3 (3.1)	5 (5.7)	0.701	0.008	0.067	0.018
Stroke at 1-year, N (%)	2 (3.2%)	1 (5.9)	2 (2)	2 (2.1)	1.000	1.000	0.802	0.868
1-year MACCE (death, non-fatal MI, non-fatal stroke), N (%)	16 (25.8)	3 (18.8)	12 (12.2)	8 (8.4)	1.000	0.092	0.029	0.097
5-year all-cause mortality, N (%)	40 (64.5)	10 (58.8)	47 (47.9)	40 (42.5)	1.000	0.152	0.038	0.047
5-year CV mortality, N (%)	33 (53.2)	4 (23.5)	21 (21.4)	17 (17.9)	0.075	0.001	0.001	0.002

LF; Low Flow, LG; Low Gradient, NF; Normal Flow, HG; High Gradient, CV; Cardiovascular, MACCE; Major Adverse Cardiovascular and Cerebrovascular Events. *p* *: *p*-value for between-group comparison.

**Table 5:** 30-Day secondary endpoint events.

	LF-LG (N = 62)	NF-LG (N = 17)	LF-HG (N = 98)	NF-HG (N = 95)	p LF-LG vs. NF-LG	p LF-LG vs. LF-HG	p LF-LG vs. NF-HG	*p* *
**30-day PPI N (%)**	31 (50)	7 (41.2)	41(41.8)	34 (35.8)	0.683	0.419	0.147	0.561
**30-day Major vascular complications N (%)**	2 (3.2)	1 (5.9)	8 (8.2)	7 (7.4)	0.660	0.294	0.353	0.755
**30-day Life-threatening/major bleeding complications N (%)**	9 (14.5)	2 (11.7)	12 (12.2)	15(15.8)	0.659	0.622	0.965	0.863

PPI; Permanent Pacemaker Implantation, LF; Low Flow, LG; Low Gradient, NF; Normal Flow, HG; High Gradient. Values are N (%). *p* *: *p*-value for between-group comparison.

**Table 6:** Multivariable Cox proportional hazards regression analysis with late (5-year) all-cause mortality as the dependent variable.

							95.0% CI for Exp(B)	
	B	SE	Wald	df	Sig.	Exp(B)	Lower	Upper
**EUROSCORE II**	−0.025	0.016	2.455	1	0.117	0.975	0.945	1.006
**CHRONIC KIDNEY DISEASE**	0.429	0.275	2.441	1	0.118	1.536	0.896	2.633
**PRIOR MI**	0.139	0.301	0.213	1	0.645	1.149	0.637	2.072
**Pre-TAVI MR ≥ moderate**	0.069	0.291	0.057	1	0.812	1.072	0.606	1.894
**Pre-TAVI TR ≥ moderate**	1.128	0.322	12.288	1	0.000	3.091	1.645	5.809
**Pre-TAVI PASP**	0.005	0.012	0.194	1	0.660	1.005	0.983	1.028
**Pre-TAVI SVi**	−0.051	0.018	8.316	1	0.004	0.951	0.918	0.984
**Post-TAVI PASP**	0.010	0.013	0.641	1	0.423	1.010	0.985	1.036
**Post-TAVI PVL ≥ moderate**	1.105	0.288	11.677	1	0.042	1.456	1.106	1.792
FLOW-GRADIENT STATE * US *			9.030	3	0.029			
**LF-HG AS**	−0.374	0.505	0.548	1	0.459	0.688	0.256	1.852
**NF-LG AS**	0.564	0.553	1.039	1	0.308	0.360	0.594	5.197
**LF-LG AS**	−1.022	0.433	5.572	1	0.018	1.757	0.154	0.841

* NF-HG AS is set as a reference. MI; Myocardial Infarction, MR; Mitral Regurgitation, TR; Tricuspid Regurgitation, PASP; Pulmonary Artery Systolic Pressure, Svi; Stroke Volume indexed, PVL; Paravalvular Leak, AS; Aortic Stenosis, LF-LG; Low Flow-Low Gradient, NF-LG; Normal Flow-Low Gradient, LF-HG; Low Flow-High Gradient, NF-HG; Normal Flow-High Gradient.

**Table 7:** Factors associated with LVEF Improvement at 1-year post-TAVI.

	Univariate Model			Multivariate Model		
	Standardized Coefficient (Beta)	R^2^	*p* Value	Standardized Coefficient (Beta)	R^2^	*p* Value
**Clinical variables**						
**Age, yrs**	−0.021	0.0005	0.856			
**Male**	0.156	0.021	0.265			
**BMI, kg/m^2^**	0.102	0.014	0.288			
**Hypertension**	−0.078	0.006	0.724			
**Diabetes**	−0.092	0.008	0.623			
**NYHA functional class III–IV**	−0.062	0.006	0.621			
**CAD**	−0.188	0.032	0.088			
**Previous MI**	−0.155	0.024	0.045	−0.193	0.036	0.054
**Previous PCI**	−0.204	0.053	0.065			
**Previous CABG**	−0.242	0.068	0.052			
**CKD**	0.102	0.009	0.355			
**PAD**	0.126	0.012	0.248			
**COPD**	−0.036	0.0008	0.726			
**EUROSCORE II**	0.074	0.009	0.273			
**Baseline echocardiographic variables**						
**LVEF, %**	−0.922	0.261	<0.001	−0.906	0.274	<0.001
**Mean gradient, mmHg**						
**AVA, cm^2^**						
**Moderate-severe MR**						
**Moderate-severe TR**						
**Stroke Volume indexed, mL/m^2^**	−0.875	0.211	<0.001	−0.898	0.211	<0.001

BMI; Body Mass Index, CABG; Coronary Artery Bypass Graft surgery, CAD; Coronary Artery Disease, CKD; Chronic Kidney Disease COPD; Chronic Obstructive Pulmonary Disease, HG; High-Gradient, LF; Low-Flow, LG; Low-Gradient, MI; Myocardial Infarction; NF; Normal Flow, NYHA; New York Heart Association, PAD; Peripheral Arterial Disease, PCI; Percutaneous Coronary Interventions, LVEF; Left Ventricular Ejection Fraction, AVA; Aortic Valve Area, MR; Mitral Regurgitation, TR; Tricuspid Regurgitation.

## Data Availability

All data presented in the study are available to the editors upon request.

## Abbreviations

AS	Aortic stenosis
AV	Aortic valve
AVA	Aortic valve area
CABG	Coronary artery bypass graft
CKD	Chronic kidney disease
COPD	Chronic obstructive pulmonary disease
CV	Cardiovascular
DSE	Dobutamine stress echocardiography
F	Flow
HG	High gradient
LF-LG	Low flow-low gradient
LVEF	Left ventricular ejection fraction
MACCE	Major adverse cardiovascular and cerebrovascular events
MG	Mean gradient
MI	Myocardial infarction
MR	Mitral regurgitation
MSCT	Multi-slice computed tomography
PAD	Peripheral artery disease
PASP	Pulmonary artery systolic pressure
PCI	Percutaneous coronary intervention
PVL	Paravalvular leak
SAVR	Surgical aortic valve replacement
SVi	Stroke volume indexed
TAVI	Transcatheter aortic valve implantation
TR	Tricuspid regurgitation
